# Age-related trends in genetic parameters for *Larix kaempferi *and their implications for early selection

**DOI:** 10.1186/1471-2156-15-S1-S10

**Published:** 2014-06-20

**Authors:** Meng Lai, Xiaomei Sun, Dongsheng Chen, Yunhui Xie, Shougong Zhang

**Affiliations:** 1 State Key Laboratory of Tree Genetics and Breeding, Research Institute of Forestry, Chinese Academy of Forestry, Beijing 100091, China

**Keywords:** *Larix kaempferi*, early selection, genetic parameters, genetic correlations, selection efficiencies

## Abstract

**Background:**

Japanese larch (*Larix kaempferi*) has been introduced in China at the end of the 19th century, and as one successful exotic species, is becoming the preferred coniferous in northern China and sub-tropical alpine region. The rotation age is about 25-28 years for *L. kaempferi *as pulpwood in Henan province. Waiting for even one-half rotation age for final evaluation will be inefficient due to accumulated testing costs and delayed return on investment, which suggests that selection at an early age is highly desirable for *L. kaempferi *improvement programs in Henan province. In this study, we determined age trends of genetic parameters and evaluated early selection efficiency for *L. kaempferi *in Henan province to find out the appropriate trait for early selection and its selection age.

**Results:**

Growth traits of 78 clones were measured periodically from age 2 to age 15 in a clonal trial of *Larix kaempferi *establishted at Son town, Henan Province. The genetic variation among clones, age-age correlations, and age trends in genetic parameters for growth traits were analyzed. Variant analysis revealed that tree height (HGT) and diameter at breast (DBH) were significant (1% level) among clones at every ages. The clonal repeatability of growth traits varied year-by-year, reaching the highest levels at different ages for different traits (0.77 at age 2 for HGT, 0.70 at age 5 for DBH and 0.66 from age 8 to age 10 for volume, respectively). The age-age genetic correlations ranged from 0.904 to 1.000 for HGT, and from 0943 to 1.000 for DBH. DBH at different ages was more genetically correlated to volume-15 than HGT. At the phenotypic level, HGT was always less correlated to volume-15 than DBH. With the estimates of efficiencies of early selection, the recommendation from present study was that the optimum age of early selection was age 2 for HGT and age 5 for DBH.

**Conclusions:**

Our study showed that there were significant (1% level) on growth traits among clones at every ages. The genetic parameters for growth traits varied from age to age. We found dual trait selection was more efficient than single trait selection for early selection.

## Background

Larch (*Larix *sp.) is one of the most valuable conifers in boreal and temperate forests as well as in mountainous regions where it is either native or introduced in artificial plantations [1]. It is of great ecological and economical importance and is highly appreciated for wood properties including high mechanical strength, attractive reddish colour and high natural durability. Japanese larch ( *Larix kaempferi*) has been introduced in China at the end of the 19^th ^century, and as one successful exotic species, is becoming the preferred coniferous in northern China and sub-tropical alpine region due to its superior performance on fast-growing at early ages, higher wood specific gravity, comparable fiber length, pest resistance and wide adaptation [2]. As a result, the area of Japanese larch plantation has been over 0.3 million hectares in China, and has been increasing at a speed of 300 thousand hectares annually.

The rotation age is about 25-28 years for *L. kaempferi *as pulpwood in Henan province. Waiting for even one-half rotation age for final evaluation will be inefficient due to accumulated testing costs and delayed return on investment, which suggests that selection at an early age is highly desirable for *L. kaempferi *improvement programs in Henan province.

Age trends for genetic parameters are crucial for developing tree breeding strategy and early selection [3]. A number of studies have documented age trends in these parameters for loblolly pine (*Pinus. taeda*) [4-9], Scots pine (*P. sylvestris*) [10-12], maritime pine (*P. pinaster*) [13], lodgepole pine *(P. contorta*) [14,15], jack pine (*P. banksiana*) [16,17,3], and Douglas-fir (*P. menziesii*) [18,19]. However, relatively few authors have addressed trends over time in genetic parameters for *L. kaempferi*. After the analyses of age trends in heritability, juvenile-mature correlations and genetic gains, Sun et al. [20] found that the most proper age for early selection was age 6, and diameter was a better predictor than height due to its genetic stability. In a clonal trail of *L. kaempferi *in northern China, Ma et al. [26] found that the Lambeth model generally fit genetic correlations well, and the highest selection efficiency for height was achieved at age 10 by using height at age 20 as selection criterion. The objectives of the study were, on the basis of a clonal trail of *L. kaempferi *that included 78 clones, (1) to determine age trends of genetic parameters, (2) to estimate age-age correlations for HGT and DBH, (3) to estimate age-age correlations for HGT and DBH with VOL-15, (4) to evaluate early selection efficiency for *L. kaempferi *in Henan province.

## Methods

### Trial description

The data were collected from a clonal trial established at Son town in Henan (34°14'N, 112°07'E), and with annual mean temperature of 8.6°C and annual rainfall of 800-1200mm. Minimum January temperature and maximum July temperature at this region were -15.5°C and 24.7°C, respectively. The soil was brown earth and pH = 6.0. 78 *L. kaempferi *clones were planted in the spring of 1998. Field design was randomized complete blocks with four replications and 4-tree plot in a spacing of 2 m × 2 m.

### Data collection

Diameter at breast (DBH) and height (HGT) were measured for all trees. HGT was measured from 2 to 15 yeas after planting, and DBH was measured from 5 to 15 years after planting. The traits analysed in this study were referred to as DBH-8, HGT-4 etc, the numbers indicating the ages. Individual tree volume (VOL in m^3^) was calculated using the following tree volume formula [22]:

(1) $$\text{VOL}=0.0000592372\times {\text{DBH}}^{1.8655726}\times {\text{HGT}}^{0.98098962}$$

### Statistical analysis

In this study, a nonlinear mixed model by using Richards growth function as basic model was constructed to fit the relationship for first-hand data of growth traits on age.

Richards growth function was as followed:

(2) $$Y=a{\left(1-{\text{e}}^{-bT}\right)}^{c}$$

Where *Y *is height (HGT) or diameter at breast (DBH), *a, b *and *c *are parameters, and *T *is the age of the trees.

Nonlinear mixed model was as followed:

(3) $$Y=\left(a+\upsilon_{L}+\upsilon_{R}\right){\left[1-e^{-\left(b+\omega_{L}+\omega_{R}\right)T}\right]}^{c}+\varepsilon$$

Where $\upsilon_{L}$ and $\upsilon_{R}$, and $\omega_{L}$ and $\omega_{R}$ are random coefficients at the clone and replication levels for *a *and *b*, respectively, and *c *was not allowed to vary randomly. The variance-covariance structures were positive-definite at both the clone $\Psi_{L}$ and replication $\Psi_{R}$ levels, and specified as:

(4) $$\Psi_{L}\text{ = }\left(\begin{array}{c} \sigma_{\upsilon L}^{2}\\ \sigma_{\upsilon \omega L}^{2} \end{array}\right)\left(\begin{array}{c} \sigma_{\upsilon \omega L}^{2}\\ \sigma_{\omega L}^{2} \end{array}\right)\;\text{and}\;\Psi_{R}\text{ = }\left(\begin{array}{c} \sigma_{\upsilon R}^{2}\\ \sigma_{\upsilon \omega R}^{2} \end{array}\right)\left(\begin{array}{c} \sigma_{\upsilon \omega R}^{2}\\ \sigma_{\omega R}^{2} \end{array}\right)$$

and distributed bivariate normally with normal random errors:

$$E\left(\begin{array}{c} \upsilon_{L}\\ \omega_{L} \end{array}\right)=0\;\upsilon_{\alpha r}\left(\begin{array}{c} \upsilon_{L}\\ \omega_{L} \end{array}\right)\text{ = }\left(\begin{array}{c} \sigma_{\upsilon L}^{2}\\ \sigma_{\upsilon \omega L}^{2} \end{array}\right)\left(\begin{array}{c} \sigma_{\upsilon \omega L}^{2}\\ \sigma_{\omega L}^{2} \end{array}\right)$$

$$E\left(\begin{array}{c} \upsilon_{R}\\ \omega_{R} \end{array}\right)=0\;\upsilon_{\alpha r}\left(\begin{array}{c} \upsilon_{R}\\ \omega_{R} \end{array}\right)\text{ = }\left(\begin{array}{c} \sigma_{\upsilon R}^{2}\\ \sigma_{\upsilon \omega R}^{2} \end{array}\right)\left(\begin{array}{c} \sigma_{\upsilon \omega R}^{2}\\ \sigma_{\omega R}^{2} \end{array}\right)$$

(5) $$\varepsilon \sim N\left(0,\sigma^{2}\right)$$

At every age, variation among clones, variance components, and genetic parameters were analyzed by analysis of variance, using a linear model [23]:

(6) $$y_{ij}=\mu +\alpha_{i}+\beta_{j}+\varepsilon_{ij}$$

where $y_{ij}$ is the performance of the *i*th clone within the *j*th block, and μ is the general mean, $\alpha_{i}$ is the effect of the *i*th clone, $\beta_{j}$ is the effect of the *j*th block, and $\varepsilon_{ij}$ is the random error.

The repeatability of clonal mean, which refers to genotypic heritability, was estimated as [23]:

(7) $$R=\sigma_{\text{c}}^{2}/\sigma_{p}^{2}=\sigma_{\text{c}}^{2}/\left(\sigma_{\text{c}}^{2}+\sigma_{e}^{2}/r\right)$$

Where *r *is the number of blocks, $\sigma_{p}^{2}$ is the phenotype variance, $\sigma_{\text{c}}^{2}$ is the variance of clone, and $\sigma_{e}^{2}$ is the residual variance.

The genetic variation coefficient was calculated using the following formula [24]:

(8) $$CVG\left(\%\right)=100\times \sqrt{\sigma_{c}^{2}}/\bar{X}$$

Where $\bar{X}$ is the trait average phenotypic mean. The equation expresses a standardized measure of the genetic variance relative to the mean of trait.

The selection gain among clones was estimated by:

(9) $$\Delta G\left(\%\right)=100\times iR\;\sigma_{p}/\bar{X}$$

Where i is the standardized selection intensity, R is the repeatability, and $\sigma_{p}^{}$ is the phenotypic standard deviation.

The phenotypic correlation of two traits (same traits at different ages were treated as different traits) was calculated as:

(10) $$r_{p}=\sigma_{p\left(xy\right)}/\sqrt{\sigma_{p\left(x\right)}^{2}\times \sigma_{p\left(y\right)}^{2}}$$

where $\sigma_{p\left(xy\right)}$ is the phenotype covariance component between traits *x *and *y*, $\sigma_{p\left(x\right)}^{2}$ is the phenotype variance component for trait *x *and $\sigma_{p\left(y\right)}^{2}$ is the phenotype variance component for trait *y*.

The genotypic correlation of two traits (same traits at different ages were treated as different traits) was calculated as [23]:

(11) $$r_{g}=\sigma_{c\left(xy\right)}/\sqrt{\sigma_{c\left(x\right)}^{2}\times \sigma_{c\left(y\right)}^{2}}$$

where $\sigma_{c\left(xy\right)}$ is the clone covariance component between traits *x *and *y*, $\sigma_{c\left(x\right)}^{2}$ is the clone variance component for trait *x *and $\sigma_{c\left(y\right)}^{2}$ is the clone variance component for trait *y*.

Efficiency of early forward selection was examined by taking growth traits at age 15(HGT-15, DBH-15, and VOL-15) as the target traits to be improved. Assuming equal intensity of selection at target and young ages, the selection efficiency (*Q*_year_), expressed as the ratio of correlated response in trait *y *at age T_2 _from a selection on trait *x *at age T_1 _per year, was calculated as [19]:

(12) $$Q_{\text{year}}=\;r_{g}\;\sqrt{R_{x}}\;T_{2}/\sqrt{R_{y}}\;T_{1}$$

Where *T*_1 _and *T*_2 _are the ages for trait *x *and target trait *y*, respectively, *r_g _*is the calculated genetic correlation between trait *x *at *T*_1 _and trait *y *at *T*_2_, and $\sqrt{R_{x}}$ and $\sqrt{R_{y}}$ are the square roots of clonal repeatability for trait *x *at *T*_1 _and trait *y *at *T*_2_, respectively. A time lag of 6 years for breeding phase was usually assumed for *L. kaempferi *in Henan province.

## Results

### Model fitting

The results of the model fitting for growth data of 78 clones are presented in table 1. The fixed parameters were significant (*p *< 0.01). The random effects of growth equation for HGT and DBH were reflected in maximum value of growth (parameter a) and growth rate (parameter b), reflecting the differences on the maximum value of growth and growth rate were significant among clones and replications. RMSE and R^2 ^were 0.5961 and 0.9543, 0.7134 and 0.9395 for HGT and DBH, respectively, and the results showed that the nonlinear mixed model fit well.

**Table 1:** The model parameters, variance components for random effects, and fit statistics for the nonlinear mixed model described in the text (Std. Dev = standard deviation). Fit statistics include the coefficient of determination (*R^2^*), root mean squared error (RMSE), Akaike information criterion (AIC), and Bayesian criterion (BIC).

Model		HGT				DBH			
Fixed effects	Parameters	Estimated values	Std. Dev	T value	P value	Estimated values	Std. Dev	T value	P value
	a	10.9349	0.2444	44.7408	< 0.01	9.0885	0.2113	43.0086	<0.01
	b	0.1436	0.0045	31.8415	< 0.01	0.2489	0.0087	28.4580	<0.01
	c	2.2299	0.0497	44.8156	< 0.01	5.3868	0.2946	18.2851	<0.01
Random effects	$\sigma_{\upsilon L}$	0.9082				0.9761			
	$\sigma_{\omega L}$	0.0113				0.0112			
	$\sigma_{\upsilon R}$	2.4430)				2.1190			
	$\sigma_{\omega R}$	0.0157				0.0292			
	$\sigma_{\upsilon \omega L}$	-0.00641				-0.00061			
	$\sigma_{\upsilon \omega R}$	-0.0240				-0.0341			
Fit statistics	R^2^	0.9543				0.9295			
	RMSE	0.5961				0.7134			
	AIC	34744.47				32408.32			
	BIC	34803.12				32464.59			

### Phenotypic variation

Mean values, ranges and F values for growth traits at different ages are presented in table 2. Over the period studied, mean values of the HGT increased from 0.50 m at age 2 to 8.25 m at age 15, the DBH increased from 1.50 cm at age 5 to 7.94 cm at age 15, and the VOL increased from 0.000415 m^3 ^at age 5 to 0.0258 m^3 ^at age 15. Meanwhile, the annual HGT increment was a mean of 0.60 m, the annual average DBH and VOL increment were 6.4 mm and 0.002538 m^3^, respectively. The results of the analysis of variance for growth traits showed that there were significant differences (1% level) on HGT, DBH and VOL among clones at every age, indicating that there were great potential for genetic improvement of growth traits among clones.

**Table 2:** Mean values, ranges and F values (**Significant at 0.01 level) for the growth traits at different ages (s.e. = standard error).

Traits	Age	Mean	Minimum	Maximum	s.e.	F Value
HGT	2	0.50	0.29	0.86	0.0121	4.35**
(m)	3	1.06	0.62	1.79	0.0244	4.20**
	4	1.73	1.02	2.88	0.0382	4.05**
	5	2.47	1.46	4.02	0.052	4.00**
	6	3.21	1.91	5.16	0.0655	3.77**
	7	3.95	2.37	6.25	0.0775	3.63**
	8	4.66	2.80	7.26	0.0884	3.49**
	9	5.33	3.22	8.18	0.0979	3.37**
	10	5.94	3.60	9.01	0.1062	3.25**
	11	6.51	3.95	9.74	0.1133	3.14**
	12	7.01	4.28	10.39	0.1196	3.05**
	13	7.47	4.57	10.96	0.1249	2.96**
	14	7.89	4.84	11.46	0.1294	2.88**
	15	8.25	5.07	11.89	0.1334	2.81**

DBH	5	1.50	0.72	2.89	0.0457	3.28**
(cm)	6	2.35	1.19	4.36	0.0662	3.26**
	7	3.26	1.71	5.82	0.0851	3.27**
	8	4.15	2.24	7.18	0.1015	3.26**
	9	4.97	2.76	8.37	0.1148	3.24**
	10	5.69	3.23	9.38	0.1254	3.20**
	11	6.32	3.65	10.21	0.1334	3.16**
	12	6.85	4.03	10.88	0.1395	3.11**
	13	7.29	4.34	11.41	0.1441	3.06**
	14	7.64	4.61	11.83	0.1476	3.01**
	15	7.94	4.83	12.16	0.1502	2.97**

VOL	5	4.15E-04	5.78E-05	1.99E-03	3.44E-05	2.74**
(m^3^)	6	1.20E-03	1.88E-04	5.32E-03	9.13E-05	2.81**
	7	2.63E-03	4.46E-04	1.07E-02	1.83E-04	2.87**
	8	4.73E-03	8.62E-04	1.80E-02	3.07E-04	2.91**
	9	7.41E-03	1.44E-03	2.67E-02	4.52E-04	2.92**
	10	1.04E-02	2.14E-03	3.58E-02	6.05E-04	2.91**
	11	1.37E-02	2.93E-03	4.48E-02	7.57E-04	2.89**
	12	1.71E-02	3.77E-03	5.34E-02	9.01E-04	2.86**
	13	2.02E-02	4.61E-03	6.11E-02	1.03E-03	2.82**
	14	2.32E-02	5.41E-03	6.80E-02	1.15E-03	2.78**
	15	2.58E-02	6.17E-03	7.39E-02	1.25E-03	2.74**

### Age trends in genetic parameters

Age trends in variance components, genetic variation coefficients (CVG), clonal repeatability (R) and genetic gains (ΔG) for growth traits are shown in table 3. Variance components for growth traits showed a clear pattern of change with time, they were increased with aging. It is evident that the variance components of HGT and DBH were higher than those of VOL for all ages. The coefficients of variation at the clonal level (table 3) were, in general, ranging between 11.47 and 18.65 percent for HGT, between 13.61 and 22.47 percent for DBH and between 34.21 and 58.33 percent for VOL. For all ages, the CVG of VOL was higher than those of HGT and DBH, and the CVG of DBH was higher than the CVG of HGT at the same age. A decreasing trend with age for growth traits was found for CVG in our studies.

**Table 3:** The variances associated with clone ($\sigma_{c}^{2}$), residual error ($\sigma_{e}^{2}$) and phenotype ($\sigma_{p}^{2}$), genetic variation coefficients (CVG), clonal repeatability (R) and genetic gains (ΔG) with 5% selection rate for the growth traits at different ages.

Traits	Age	$\sigma_{c}^{2}$	$\sigma_{e}^{2}$	$\sigma_{p}^{2}$	CVG (%)	R	ΔG (%)
HGT	2	0.0087	0.0104	0.0113	18.65	0.77	33.77
	3	0.0353	0.0441	0.0463	17.72	0.76	31.92
	4	0.0855	0.1121	0.1135	16.90	0.75	30.26
	5	0.1586	0.2117	0.2102	16.12	0.75	28.65
	6	0.2445	0.3540	0.3335	15.40	0.73	27.27
	7	0.3396	0.5168	0.4688	14.75	0.72	25.90
	8	0.4353	0.6978	0.6098	14.16	0.71	24.68
	9	0.5262	0.8893	0.7485	13.61	0.70	23.54
	10	0.6094	1.0832	0.8802	13.14	0.69	22.56
	11	0.6839	1.2760	1.0029	12.70	0.68	21.64
	12	0.7494	1.4646	1.1156	12.35	0.67	20.88
	13	0.8057	1.6447	1.2169	12.02	0.66	20.17
	14	0.8534	1.8150	1.3072	11.71	0.65	19.52
	15	0.8947	1.9749	1.3884	11.47	0.64	18.99

DBH	5	0.1136	0.1991	0.1634	22.47	0.70	38.65
	6	0.2368	0.4191	0.3416	20.71	0.69	35.57
	7	0.3928	0.6924	0.5659	19.23	0.69	33.04
	8	0.5576	0.9855	0.8040	17.99	0.69	30.91
	9	0.7116	1.2699	1.0291	16.97	0.69	29.12
	10	0.8426	1.5308	1.2253	16.13	0.69	27.60
	11	0.9484	1.7586	1.3881	15.41	0.68	26.28
	12	1.0300	1.9544	1.5186	14.82	0.68	25.17
	13	1.0905	2.1198	1.6205	14.32	0.67	24.24
	14	1.1361	2.2572	1.7004	13.95	0.67	23.53
	15	1.1673	2.3724	1.7604	13.61	0.66	22.86

VOL	5	5.86E-08	1.34E-07	9.21E-08	58.33	0.64	95.99
	6	4.18E-07	9.28E-07	6.50E-07	53.88	0.64	89.13
	7	1.72E-06	3.68E-06	2.64E-06	49.87	0.65	83.04
	8	4.85E-06	1.02E-05	7.40E-06	46.56	0.66	77.76
	9	1.05E-05	2.18E-05	1.60E-05	43.73	0.66	73.20
	10	1.87E-05	3.93E-05	2.85E-05	41.58	0.66	69.45
	11	2.93E-05	6.20E-05	4.48E-05	39.51	0.65	65.92
	12	4.12E-05	8.87E-05	6.34E-05	37.54	0.65	62.44
	13	5.37E-05	1.18E-04	8.32E-05	36.28	0.65	60.13
	14	6.62E-05	1.48E-04	1.03E-04	35.07	0.64	57.95
	15	7.79E-05	1.79E-04	1.23E-04	34.21	0.64	56.24

The clonal repeatability ranged from 0.64 to 0.77 for HGT with the highest occurring at age 2, from 0.66 to 0.70 for DBH with the highest occurring at age 5, and from 0.64 to 0.66 for VOL with the highest occurring from age 8 to age 10. On the whole, the clonal repeatability of HGT and DBH were decreased with ageing, as the clonal repeatability of VOL increased from 0.64 at age 5 to 0.66 at age 8, keep it at this level until age 10, and then decreased again. Time trends in genetic gains for grow traits among clones selection, with 5% selection rate (or intensity = 2.063), showed that the greatest gains were reached at age 2 for HGT and age 5 for both DBH and VOL.

Estimated age-age genetic correlations between HGT at different ages and HGT-15 varied from 0.904 to 1.000 (table 4). The corresponding estimated age-age phenotypic correlations ranged from 0.887 to 1.000. Age-age genetic correlations for DBH varied from 0.943 to 1.000. For all ages, the DBH were more genetically correlated to DBH-15 than HGT to HGT-15. Phenotypic correlations for DBH ranged from 0.905 to 1.000, and were generally lower than corresponding genetic correlations estimates for all ages. As the age difference decreased, both the age-age genetic and phenotypic correlations for HGT or DBH increased.

**Table 4:** Estimated genetic correlations (r_g_) and phenotypic correlations (r_p_), for height at age 15 (HGT-15) with various heights, and diameter at age 15 with various diameters.

Age	HGT		DBH	
	r_g_	r_p_	r_g_	r_p_
2	0.904	0.887**	-	-
3	0.917	0.906**	-	-
4	0.928	0.920**	-	-
5	0.939	0.934**	0.943	0.905**
6	0.950	0.947**	0.956	0.926**
7	0.960	0.958**	0.966	0.944**
8	0.970	0.969**	0.976	0.960**
9	0.978	0.977**	0.983	0.973**
10	0.985	0.985**	0.989	0.983**
11	0.990	0.990**	0.994	0.990**
12	0.995	0.995**	0.997	0.995**
13	0.997	0.997**	0.999	0.998**
14	0.999	0.999**	1.000	0.999**
15	1.000	1.000**	1.000	1.000**

**Significant at 0.01 level.

Estimated of genetic correlations and phenotypic correlations between VOL-15 and various HGT or DBH are listed in table 5. The genetic and phenotypic correlations involving VOL-15 and various HGT increased with ageing, and the values ranged from 0.849 to 1.000. The same trend was observed for genetic and phenotypic correlations between VOL-15 and various DBH (rang 0.897-1.000). It is evident that the genetic correlations between DBH and VOL-15 were stronger than corresponding correlations with HGT at the same age. At the phenotypic level, HGT was always less correlated to VOL-15 than DBH.

**Table 5:** Estimated genetic correlations (r_g_) and phenotypic correlations (r_p_), for tree volume at age-15 (VOL-15) with various heights or diameters.

Age	HGT		DBH	
	r_g_	r_p_	r_g_	r_p_
2	0.882	0.849**	-	-
3	0.890	0.867**	-	-
4	0.917	0.879**	-	-
5	0.919	0.889**	0.923	0.897**
6	0.926	0.899**	0.935	0.912**
7	0.942	0.908**	0.943	0.925**
8	0.944	0.915**	0.952	0.936**
9	0.955	0.922**	0.960	0.945**
10	0.955	0.927**	0.964	0.951**
11	0.964	0.930**	0.965	0.955**
12	0.964	0.932**	0.971	0.957**
13	0.964	0.933**	0.971	0.958**
14	0.969	0.934**	0.972	0.958**
15	0.969	0.934**	0.972	0.958**

**Significant at 0.01 level.

### Efficiencies of early selection

**Figure 1:**
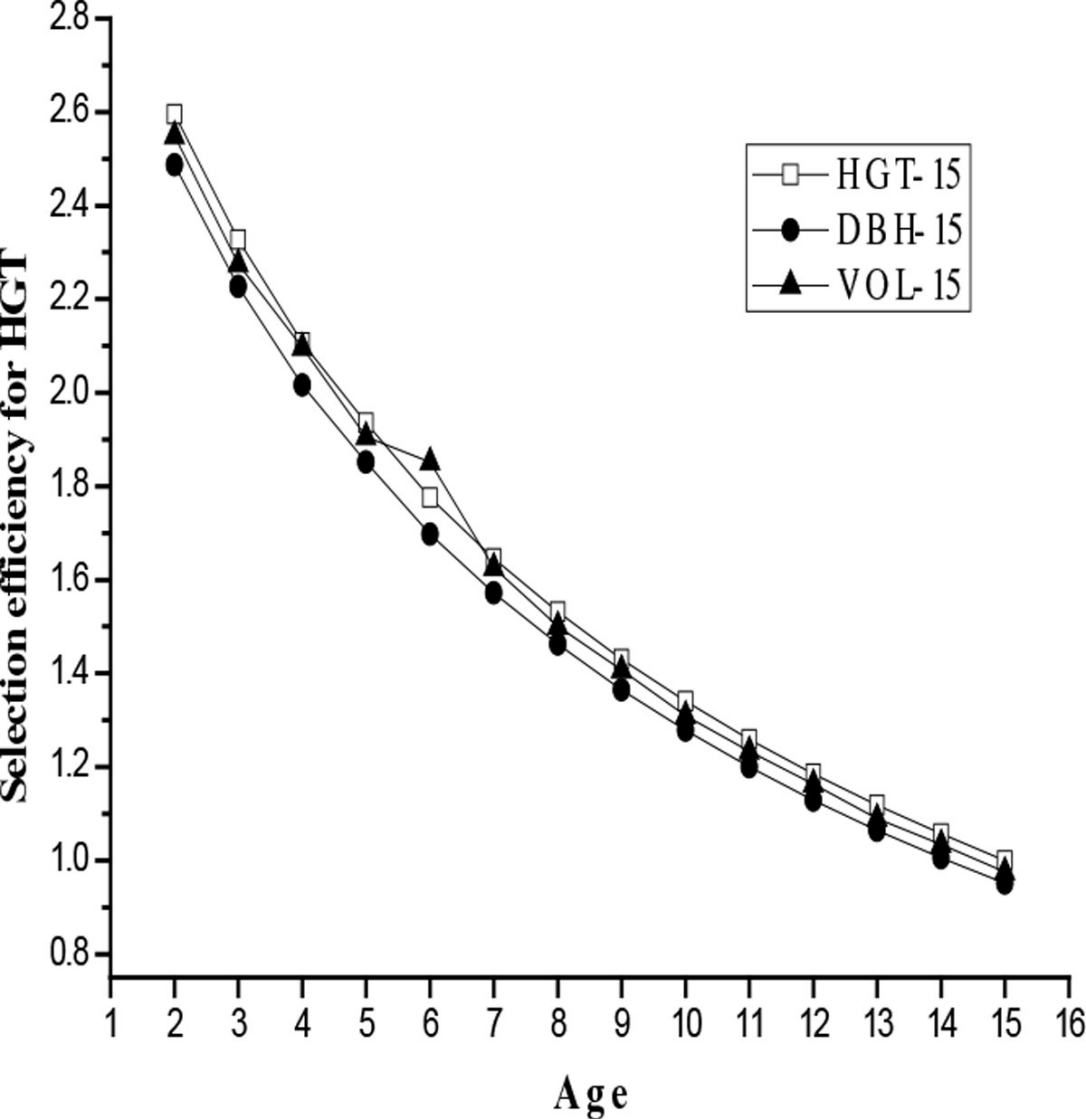
**Selection efficiency (*Q*_year_) for HGT, expressed as the ratio of correlated response in growth traits at age 15 from a selection on various heights**.

**Figure 2:**
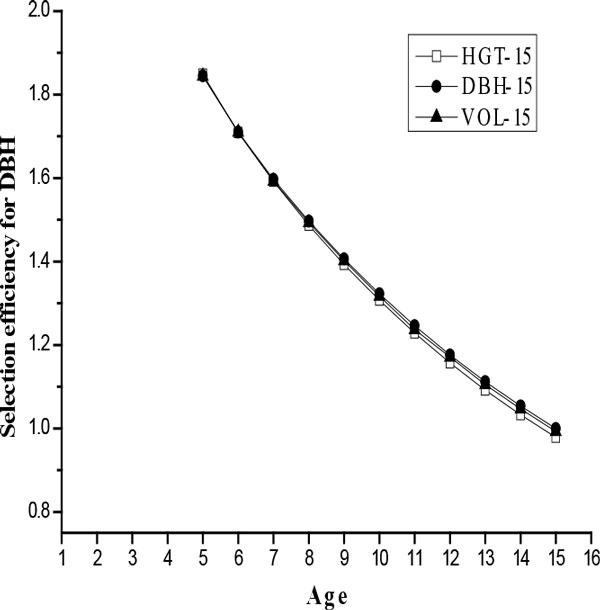
**Selection efficiency (*Q*_year_) for DBH, expressed as the ratio of correlated response in growth traits at age 15 from a selection on various diameters**.

The efficiencies of early selection (*Q*_year_) in growth traits at age 15, through early selection on various HGT and DBH, are shown in Figure 1 and 2, respectively. Although the magnitudes of the selection efficiency varied with time, study indicated that selection made at the first measurement year would be more efficient than direct growth traits selection at age 15. That is, indirect selection on HGT-2 and DBH-5 could be expected to produce the most gain per year in growth traits at age-15 compared with direct selection on HGT and DBH themselves.

## Discussion

The variance components, genetic variation coefficients (CVG), clonal repeatability (R) and genetic gains (ΔG) for growth traits are dynamic during whole period of tree growth and show some certain rules. An increasing trend with age of variance components for growth traits was found in this study, this trend in variance components was similar to those found in Norway spruce [25] and Scots pine [10].

Grasping the age trends of genetic variation coefficients, clonal repeatability and genetic gains are very important for determining the appropriate early selection time and estimating the effects of early selection [26]. The coefficients of genetic variation (CVG), that is, the genetic variance standardized to trait mean, is considered to be the most suitable parameter for comparisons of genetic variation and the ability to respond to natural or artificial selection [27]. In the present study, the CVG of VOL was higher than the CVG of HGT or DBH at the same age, agreeing with previous study of jack pine which revealed that the CVA (additive genetic coefficient of variation) for volume, at one-half rotation age was almost 2-3 times higher as that for height [3]. Besides, the CVG of DBH was higher than the CVG of HGT at the same age, indicating that the scope for selection among clones of DBH is larger than that for HGT. The CVG for growth traits decreased with ageing, with regarded to the CVA, similar trend has been reported in other studies [10,11,28,29].

Clonal repeatability estimates for growth traits in this study ranged from 0.64 to 0.77, which means that variation in growth traits of *L. kaempferi *were controlled genetically at medium or upwards level. As a whole, the clonal repeatability of HGT decreased with ageing, agreeing with previous study by Vasquez and Dvorak [30]. Vasquez and Dvorak [30] investigated the trend of heritability for height in tropical pine species during first 8 years of growth, and found that in *P. tecunumanii *and *P. chiapensis *the heritability of height was decreased with aging. However, Xiang et al. [8] found that the general trend of heritability estimates was increasing over time. Danjon [31] found that the heritability of height in *P. pinaster *increased after 5 years and remained fairly constant after age 10 years. The clonal repeatability of DBH followed a similar trend over time as HGT, which decreased with increasing age, in agreement with former finding in lodgepole pine [15]. Nevertheless, with regard to the heritability in other studies, Jonson et al. [18] found that the heritability of diameter showed an increase with aging for Douglas-fir while the heritability of height was mostly stable over time. Xiang et al. [8] reported that the heritability of diameter increased from age 4 to age 8. The clonal repeatability of VOL was mostly stable over time, ranging from 0.64 to 0.66, the values of clonal repeatability for VOL were a few points lower than those of HGT and DBH, reflecting the influence of HGT and DBH on VOL.

Age-age genetic correlations for HGT or DBH in this study were impressive high, and the results suggest that the genes involved in early age HGT or DBH growth appear to be similar to those affecting the same trait at age 15. The age-age genetic correlations for DBH were stronger than those of HGT for all ages, differed from those of Gwaze and Bridgewater [6] who revealed that at young ages (<8 years) height was more genetically correlated to height at 25 years than diameters to diameter at 25 years. Dean and Stonecypher [19] found that, from age 5 to age 10, the genetic correlations involving height and height-17 were stronger than the genetic correlations between diameter and diameter-17 at the same age.

Age-age genetic correlations between various HGT or DBH and VOL at age 15 (VOL-15) were strong. In general, the age-age genetic correlations presented here are similar to other findings in Douglas-fir [18], Norway spruce [25]. The age trend of age-age genetic correlations between various HGT and VOL-15 was similar to the age trend of genetic correlations between various DBH and VOL-15, which increased with ageing. The results in this study were different from the observations in loblolly pine, in which Xiang et al. [8] reported that the shape of the trend curve over time for genetic correlations of trait height with age-8 volume was different than the corresponding curve for genetic correlations of trait diameter with age-8 volume. Age-age genetic correlations between the various HGT and VOL-15 were lower than those between DBH and VOL-15 for all ages. Our results in agreement with those of Li and Mckeand [32] who found that genetic correlations between various heights and volume at age 20 were always lower than those of between the various diameters and volume at age 20. However, Gwaze and Bridgewater [6] found that at young ages (< 7 years) height was more genetically correlated to volume at 25 years than diameter.

It is believed that the age when efficiency of early selection reached the maximum value was the optimum age for early selection [33]. In this study we have used growth traits at age 15 as the selection criterion, results in the present study indicate that early selection for *L. kaempferi *in Henan province could be effective. High genetic correlations between growth traits at age 15 and various HGT or DBH should explain the observation. In our studies, the optimum selection age for HGT using growth traits at age 15 as selection criterion (age 2) was 3 years lower than those for DBH using growth traits at age 15 as selection criterion (age 5). Although the highest selection efficiency was achieved at the first measurement year, i.e., age 2 for HGT and age 5 for DBH, the true optimal age could potentially be even earlier. Optimum selection age for DBH in this study was slightly lower those estimated by Sun et al. [20] and Ding et al. [34] (6-7 years for family selection). A latter early selection age for HGT of *L. kaempferi *was found in the study of Ma et al. [21], in which the optimum age of early selection for HGT was age 10 in northern of China.

Some researchers thought the superiority of height for early selection was due to its higher heritability than diameter [35-37]. However, Li and Mckeand [32] inferred that optimum selection age for diameter was likely to be lower than that of height given the higher age-age correlations and the comparable heritability estimates, and thus diameter should be more effective than height as the trait for early selection. In this study, the efficiencies (Q_year_) of early selection on HGT at young ages (< 10 years) in terms of indirect gains per year in vol-15 were higher than those for DBH, suggesting that HGT might be a better early selection criterion than DBH. However, with the analyses of age trends for HGT and DBH in genetic parameters, we found DBH was a better predictor than HGT. These results indicate that dual trait selection might be more reliable than single trait selection for early selection, agreeing well with results for China fir (*Cunninghamia. Lanceolata*) published elsewhere [21,38].

The strength of this study is that the population sample size was large (78 clones) and a nonlinear mixed model was used to fit the relationship for first-hand data of HGT and DBH on age, therefore allowed reasonably precise genetic statistics and realistic predictions of rotation age gains. However, the study is limited by the fact that it was established at only one site. The genetic parameters and age-age correlations have been shown to differ among sites or geographic regions [3,4].

## Conclusions

In conclusion, there were significant differences (1% level) on growth traits among clones at every ages. The genetic parameters for growth traits varied from age to age. The genetic correlations involving VOL-15 and various HGT or DBH increased with ageing, and HGT was always less correlated to VOL-15 than DBH at the genetic level. Using growth traits at age 15 as the selection criterion, the highest selection efficiency was achieved at the first measurement year, thus the optimal selection age was age 2 for HGT and age 5 for DBH, and dual trait selection was more efficient than single trait selection for early selection.

## Competing interests

The authors declare that they have no competing interests.

## Authors' contributions

ML conducted the study and wrote the manuscript. XM Sun carried out the critical reading and grammatical correction of manuscript. SG Zhang was mainly responsible for who gained the fund providing the study need. DS Chen and YH Xie participated in discussions and helped to draft the manuscript. All authors read and approved the final manuscript.

## Declarations

Publication charges for this work were funded by a grant from the National Science & Technology Pillar Program in the Twelfth Five-Year Plan Period of China (2012BAD01B01).

This article has been published as part of *BMC Genetics *Volume 15 Supplement 1, 2014: Selected articles from the International Symposium on Quantitative Genetics and Genomics of Woody Plants. The full contents of the supplement are available online at http://www.biomedcentral.com/bmcgenet/supplements/15/S1.
